# Stonewall and Brickwall: Two Partially Redundant Determinants Required for the Maintenance of Female Germline in *Drosophila*

**DOI:** 10.1534/g3.118.200192

**Published:** 2018-04-24

**Authors:** Vallari Shukla, Neena Dhiman, Prajna Nayak, Neelesh Dahanukar, Girish Deshpande, Girish S. Ratnaparkhi

**Affiliations:** * Department of Biology, Indian Institute of Science Education & Research, Pune 4111008, India; † Department of Molecular Biology, Princeton University, Princeton, NJ 08544

**Keywords:** MADF, BESS, Gene Duplication, Sub-functionalization, Germline Stem Cell, Stonewall, redundancy, robust

## Abstract

Proper specification of germline stem cells (GSCs) in *Drosophila* ovaries depends on niche derived non-autonomous signaling and cell autonomous components of transcriptional machinery. Stonewall (Stwl), a MADF-BESS family protein, is one of the cell intrinsic transcriptional regulators involved in the establishment and/or maintenance of GSC fate in *Drosophila* ovaries. Here we report identification and functional characterization of another member of the same protein family, CG3838/ Brickwall (Brwl) with analogous functions. Loss of function alleles of *brwl* exhibit age dependent progressive degeneration of the developing ovarioles and loss of GSCs. Supporting the conclusion that the structural deterioration of mutant egg chambers is a result of apoptotic cell death, activated caspase levels are considerably elevated in *brwl*^-^ ovaries. Moreover, as in the case of *stwl* mutants, on several instances, loss of *brwl* activity results in fusion of egg chambers and misspecification of the oocyte. Importantly, *brwl* phenotypes can be partially rescued by germline specific over-expression of *stwl* arguing for overlapping yet distinct functional capabilities of the two proteins. Taken together with our phylogenetic analysis, these data suggest that *brwl* and *stwl* likely share a common MADF-BESS ancestor and they are expressed in overlapping spatiotemporal domains to ensure robust development of the female germline.

Myb/SANT (**S**wi3, **A**da2, **N**-CoR, **T**FIIB)-like domain [InterPro, (Finn *et al.* 2017); IPR001005] protein family is comprised of transcriptional regulators that are involved in a variety of functions in diverse organismal contexts. Consistent with their involvement in gene regulation, a few of these proteins have been shown to bind to naked DNA to modulate transcription of individual genes. Alternatively they can also influence a group of genes simultaneously by regulating chromatin remodeling (Boyer *et al.* 2004). The *Drosophila melanogaster* genome consists of fifty-five Myb/SANT like in Adf (MADF) genes (Shukla *et al.* 2014). In flies, MADF domain containing genes have been implicated in critical biological processes such as hybrid incompatibility (Brideau *et al.* 2006; Maheshwari *et al.* 2008), transposition (Casola *et al.* 2007) and transcriptional regulation (Ratnaparkhi *et al.* 2008; Cutler *et al.* 1998; Timmerman *et al.* 2013). A subgroup of the MADF family is the MADF-BESS family, which consists of sixteen proteins that contain both, a N-terminal MADF and C-terminal BEAF, Su-Var (3–7), Stonewall (BESS) domain (Bhaskar and Courey 2002). The BESS domain, like MADF, may also be a modified SANT domain (Cutler *et al.* 1998; Bhaskar and Courey 2002). The MADF domain is a DNA binding domain while the BESS domain is thought to be a protein-protein interaction domain (Bhaskar and Courey 2002; Maheshwari *et al.* 2008).

The MADF-BESS family in *Drosophila* has been proposed as an example of a gene family that has duplicated and expanded (Lander *et al.* 2001; Shukla *et al.* 2014) over 40 million years (My) ago and has in the intervening period sub-functionalized (Shukla *et al.* 2014). We have been exploring the redundancy and sub-functionalization within this family using molecular genetic analysis (Shukla *et al.* 2014). In an earlier study, we reported that three members of the family (*hinge1*, *hinge2 and hinge3*) show overlapping functions during development of the wing-hinge, primarily by negative regulation of Wingless (Wg) expression (Shukla *et al.* 2014). Moreover, RNAi mediated knockdowns of all sixteen members in different tissues of the body suggested that different subsets of genes function in distinct spatiotemporal domains. For example, only *Dlip3*, but not any other MADF-BESS gene, is important for eye development (Duong *et al.* 2008) while *Coop* (Song *et al.* 2010) is involved in patterning of the wing by regulating Wingless signaling.

*Drosophila* gene *stwl* (Clark and McKearin 1996) (McKearin 1997) encodes for a heterochromatin-associated protein belonging to the MADF-BESS family (Delattre *et al.* 2004; Delattre *et al.* 2000). Aside from being the largest (1037 amino acids) member of this family, it also contains a modified SANT domain (Boyer *et al.* 2004) and has been implicated in regulating chromatin compaction (Yi *et al.* 2009). Functional characterization of Stwl protein has suggested its possible involvement during position-effect variegation (PEV) similar to Su(var)3-7. Furthermore, it is also thought that Stwl achieves this by affecting methylation of Lys residues in Histone tails (Yi *et al.* 2009). As would be expected of a pleiotropic chromatin regulator, mutations in *stwl* display phenotypes in distinct and seemingly unrelated developmental contexts including wing patterning (Shukla *et al.* 2014; Brun *et al.* 2006), DNA damage response (Yi *et al.* 2009) and, female germline development. (Clark and McKearin 1996) (Akiyama 2002).

*stwl* function seems to manifest at a number of different stages of ovarian development. Immunostaining analysis with anti-Stwl antibodies indicated that Stwl protein is nuclear protein and is specifically detected in the GSCs and their progeny including cystoblasts and cystocytes. Accordingly, Stwl appears to be required in the germline stem cell (GSC) lineage for GSC maintenance as loss of *stwl* activity leads to corresponding loss of GSCs (Clark and McKearin 1996; Maines *et al.* 2007; Akiyama 2002). Interestingly requirement of Stwl appears to be specific to GSCs alone as GSCs undergo precocious differentiation in the absence of *stwl*, however differentiated population *i.e.*, cystocytes, marked by the presence of fusome, are relatively unaffected. Furthermore, oocyte specification is also defective in *stwl* ovaries resulting in excess number of cells that retain oocyte determinants such as Orb protein (Clark and McKearin 1996). Moreover, a fraction of *stwl* cysts undergo an extra round of division (Clark and McKearin 1996). Although many of the phenotypic consequences due to loss of *stwl* function are readily apparent, they are partially penetrant and are also sensitive to external conditions such as temperature. The variability of *stwl* phenotypes led Clark & McKearin to speculate that other proteins might partially substitute for *stwl* function. Thus, they in fact, predicted that ‘*Partial functional redundancy for Stwl protein might account for the variable severity of stwl phenotypes*’ (Clark and McKearin 1996).

Here we have tested this prediction systematically. The availability of TRiP RNA interference lines (Ni *et al.* 2009; Ni *et al.* 2011; Perkins *et al.* 2015) and germline specific UAS/Gal4 strains (Staller *et al.* 2013; Rørth 1998) allow for efficient maternal knockdown. Using this strategy, we searched for another member of MADF-BESS family that potentially resembles *stwl*. Based on female germline specific knockdown and subsequent phenotypic analysis, we have uncovered CG3838, a member of the MADF-BESS family, that has germline-specific functions. Further supporting the claim, transposon insertion mutants spanning the locus are viable but only semi-fertile and fertility of adults deteriorates in an age dependent manner. Moreover, ovaries from mutant females show GSC lineage specific defects. The loss of function phenotypes of *CG3838* and *stwl* thus seem to overlap. Confirming the assertion that the two proteins are partially redundant, Stwl over-expression in the GSC lineage is sufficient to rescue several, but not all, aspects of loss of *CG3838* function. Based on our analysis, *stwl* and *CG3838*, now christened *brickwall (brwl)*, seem to retain overlapping functions which may reflect the presence of an ancestral gene that existed over 40 My ago.

## Materials & Methods

### Drosophila husbandry

All flies were raised at 25° in standard corn meal agar. Crosses were set up at 25° and 29°. The females of the F1 progeny were dissected for the phenotypes in all cases.

### Fly Lines

For ovarian somatic and maternal knockdowns, VALIUM10, VALIUM20 and VALIUM22 Transgenic RNAi Project lines procured from Bloomington Drosophila Stock Center (BDSC) were used (Ni *et al.* 2009)(Perkins *et al.* 2015) in this study. VALIUM lines used have been listed in Table 1. Here, we have used notations V10/V20/V22 to indicate a VALIUM10/VALIUM20 or VALIUM22 line respectively. For example, *UAS-CG3838 RNAi* (V22) is a VALIUM22 while *UAS-CG3838* RNAi (V10) is a VALIUM10 line. Lines expressed in the ovary include (UAS-gene name, (Source)), *UAS-CG13204* (DPiM), *UAS-CG3838* (FlyORF), *UAS-stwl* (DPiM), *UAS-CG4404* (FlyORF), *UAS-CG8359 (* FlyORF), *UAS-Coop* (FlyORF), *UAS-CG3919* (FlyORF). DPiM (*Drosophila* Protein Interaction Map), indicates use of C-terminal FLAG-HA tagged DNA clones from the DPiM (Guruharsha *et al.* 2011) project, procured from *Drosophila* Genomic Resource Centre (DGRC), Indiana and then injected in the *Drosophila* Transgenic Facilty (http://www.ccamp.res.in/Fly-facility) at NCBS Bangalore. FlyORF lines (https://flyorf.ch/) are from the Zurich FlyORFeome project (Bischof *et al.* 2013). The expression of Brwl in the GSC lineage was monitored by imaging a CG3838:GFP.FTBP fusion available in BDSC (#56150). The fly line was a Bac construct created as part of the modERN project (Flybase FBrf0225001; Personal Communication by A. Victorsen and K. White 2014). Daughters against *Dpp (dad)-lacZ* expression (Song *et al.* 2004) was monitored to mark GSCs. The expression of Stwl was monitored using Stwl:GFP protein trap line procured from FlyTrap (Kelso *et al.* 2004).

**Table 1:** Summary of a targeted screen setup to evaluate germline and somatic roles for MADF-BESS genes. RNAi lines from the Harvard *in-vivo* fly RNAi project (*TRiP*; *https://fgr.hms.harvard.edu/fly-in-vivo-rnai*) (Ni *et al.* 2009; Ni *et al.* 2011) were used for the experiments. VALIUM10 (V10), VALIUM20 (V20) and VALIUM22 (V22) lines were procured from the *Bloomington Drosophila Stock Centre* (BDSC) and crossed with *nos-Gal4:VP16* (#4937) and *c587-Gal4* (#67747). Experiments were done both at 25 °C and 29 °C. Data shown is for the 25 °C cross, which was similar to the results obtained at 29 °C. The data for maternal knockdown using both *MTD-Gal4* and *nos-Gal4;UAS-Dicer2* has been taken from Suppl. Table of (Yan *et al.* 2014)

*GENE*	*Bloomington Line*	*nos-Gal4 (Germline)*	Yan *et al.* (2014) *(Germline)*	*c587-Gal4 (Somatic)*
*CG8119*	57761 (V20)	-*^a^*	—	Normal
*CG8119*	38893 (V22)	Normal*^b^*	Normal	—
*Adf1/ CG15845*	28680 (V10)	—	Normal	Normal
*hng2/ CG8539*	58149 (V20)	Normal	—	Normal
*CG30403*	57286 (V20)	Normal	—	Normal
*CG3919*	33355 (V20)	Normal	Normal	Normal
*CG4404*	62415 (V20)	—	—	Normal
*CG4404*	50527 (V22)	Normal	—	—
*hng1/ CG9437*	26754 (V10)	—	Normal	Normal
*hng3/ CG13987*	29613 (V10)	—	Normal	—
*hng3/ CG13987*	42765 (V20)	Normal	—	Normal
*dlip3/ CG12767*	27067 (V10)	—	—	—
*CG11723*	29349 (V10)	—	Normal	Normal
*CG11723*	42514 (V20)	Normal	—	—
*Coop/ CG1621*	29350 (V10)	—	Normal	Male pupal lethal
*CG45071*	—	—	—	—
*brwl/ CG3838*	36785 (V22)	Phenotype in ovary seen in flies; 7 days post-eclosion	Normal	Normal
*brwl/ CG3838*	31922 (V10)	Normal	—	Normal
*CG6276*	62234 (V20)	—	Normal	Normal
*CG6276*	38189 (V22)	Normal	Normal	—
*CG13204*	31919 (V10)	—	—	Normal
*CG13204*	42806 (V22)	Normal	—	—
*Stwl/ CG3836*	35415 (V22)	Strong phenotype in ovary; Seen in female flies immediately after eclosion	No Eggs	—

a ‘Normal’ indicates a normal ovary without any fusion or degeneration related phenotypes in ovarioles from 10 and 18 day old females. We do see minor follicle phenotypes, such as layering, in some of the crosses but these are distinct from the phenotypes seen in *brwl* mutants. Also seen are significant variations in number of animals emerging from each cross.

b ‘-’ indicates that the experiment was not performed. Experimental data in the table represents collated data for 10 and 18 day old females.

### Gal4 drivers

*nanos(nos)*-Gal4:VP16, which expresses in the GSCs and cysts in the germarium (BDSC 4937), was the primary Gal4 used for all the experiments. Other Gal4 drivers used included maternal *α-tubulin*-Gal4:VP16 (MAT-Gal4, expresses in the germline, but not the GSCs), *c587-Gal4* (BDSC 67747, expresses in most somatic cells of the ovary), *Traffic Jam (TJ)-Gal4* (Subset of follicle cells), *hedgehog (hh)-Gal4* (cap cells) and *GR1-Gal4* (BDSC 36287, expresses in the follicle cells) (Hartman *et al.* 2015; Decotto and Spradling 2005; Jin *et al.* 2013).

### Screening MADF-BESS genes with somatic and maternal drivers

F1 Ovaries were screened for phenotypes using *nos-Gal4* as a maternal driver and *c587-Gal4* as a somatic driver. For the screen, 5 F1 females were dissected at 3, 10 and 18 days, ovaries stained with Phalloidin and DAPI and visualized under an epifluorescence microscope. ∼150 ovarioles were examined by eye for each time point and representative images were stored. V10 lines were tested with the *c587-Gal4*, V20 lines tested with both somatic (*c587-Gal4*) and germline (*nos-Gal4*) drivers and V22 lines were tested only with *nos-Gal4*.

### Transposon insertion Lines

*brwl^KG00824^* (BDSC 12901) is *P*-element insertion line in the 5′ of *CG3838* and is a part of BDGP Gene Disruption Project collection (Bellen *et al.* 2004). *brwl^MI054561^* (BDSC 42326) is Minos-element insertion line with the Minos cassette inserted in the 3′ of CG3838 and is the part of transposon Minos-mediated integration cassette (MiMic) collection (Venken *et al.* 2011). Here, these are referred to in short, as *brwl*^KG0^ and *brwl*^MI0^.

### Immunostaining and Imaging

Adult ovaries were dissected in chilled 1X PBS and fixed with 4% paraformaldehyde in 1X PBS with 0.3% Triton-X for 20 min at room temperature. They were blocked in 2% BSA, and 0.3% Triton for in 1XPBS for 1 hr; incubated with the primary antibody overnight at 4°; washed 4 times for 10 min in 1X PBS containing 0.1% Triton X and incubated with the appropriate fluorescent secondary antibody for 1 hr at room temperature in the dark. The ovaries were then washed and mounted in Antifade (P7481, Themo-Fisher Scientific), DAPI (D1306, Themofisher Scientific) was used to stain the nuclei. Primary antibodies used are mouse anti-alpha spectrin 3A9 (1:50), mouse anti-orb 4H8 (1:10), rat anti-vasa (1:50), anti-hnt 1G9 (1:10), anti B-gal 40-1a (1:10) from DSHB, rabbit anti-phospho-histone S10 (#9701; 1:150), rabbit cleaved Caspase-3 (#9661S; 1:150) from Cell signaling, rabbit anti-cyclin E (1:100) from Cell signaling, rabbit anti-GFP from Sigma and Chicken Anti-GFP (#A10262, 1:500) from Thermo-Fisher Scientific. Mouse anti-C3G (1:500) was kindly provided to us by Prof. Scott Hawley. All secondary antibodies used were from Invitrogen Molecular Probes. Phalloidin 568 and 488 (Invitrogen Molecular Probes) were used to stain actin at a dilution of 1:100. Images were taken on Zeiss 710 LSM confocal microscope and Leica SP8 confocal microscope at 40X and 63X and subsequently processed using Image J software. GraphPad Prism 6 was used for making graphs and for statistical analysis.

### Phylogenetic Analysis

The coding sequence of MADF and the BESS domains from *brwl* and *stwl* genes in 12 *Drosophila* species was predicted using NCBI Conserved Domain (Marchler-Bauer *et al.* 2015). The sequences were aligned using MUSCLE (Edgar 2004) implemented in MEGA7.0 (Kumar *et al.* 2016). Pairwise raw genetic distances were calculated in MEGA7.0 (Kumar *et al.* 2016). Sequences were partitioned into three codon positions to create a full partition and then the greedy strategy (Lanfear *et al.* 2012) implemented in IQ-Tree (Nguyen *et al.* 2015) was used to find the right partitioning scheme based on minimum Bayesian Information Criterion (BIC) (Nei M 2000) (Schwarz 1978). Pairwise codon substitution pattern (ω = dN/dS) was determined in DnaSP (Librado and Rozas 2009). Each domain was tested for recombination and appropriate codon substitution model in Datamonkey (Delport *et al.* 2010). Codon wise accumulation of synonymous and non-synonymous codons was analyzed using REL method (Pond *et al.* 2005) implemented in Datamonkey (Delport *et al.* 2010).

### Data and Reagent availability

Fly lines are available on request. The authors affirm that all data necessary for confirming the conclusions of the article are present within the article, figures, and tables. Supplemental material available at Figshare: https://doi.org/10.25387/g3.6114977.

## Results

### A targeted screen to uncover MADF-BESS genes required in the GSC lineage

As previously reported (Clark and McKearin 1996; Akiyama 2002), reducing *stwl* function in the female germline led to ovarian phenotypes. This phenotype is replicated by driving *stwl RNAi (V20)* in the germline using *nos-Gal4* [See Suppl. Figure 1 and (Yan *et al.* 2014)]. Consistent with the previous reports the phenotypic consequences due to loss of *stwl* in adult ovaries were severe resulting in very small ovarioles that degenerated within days. Earlier studies have also demonstrated that Stwl is expressed in the GSCs and cysts in the germarium (Clark and McKearin 1996).

**Figure 1:**
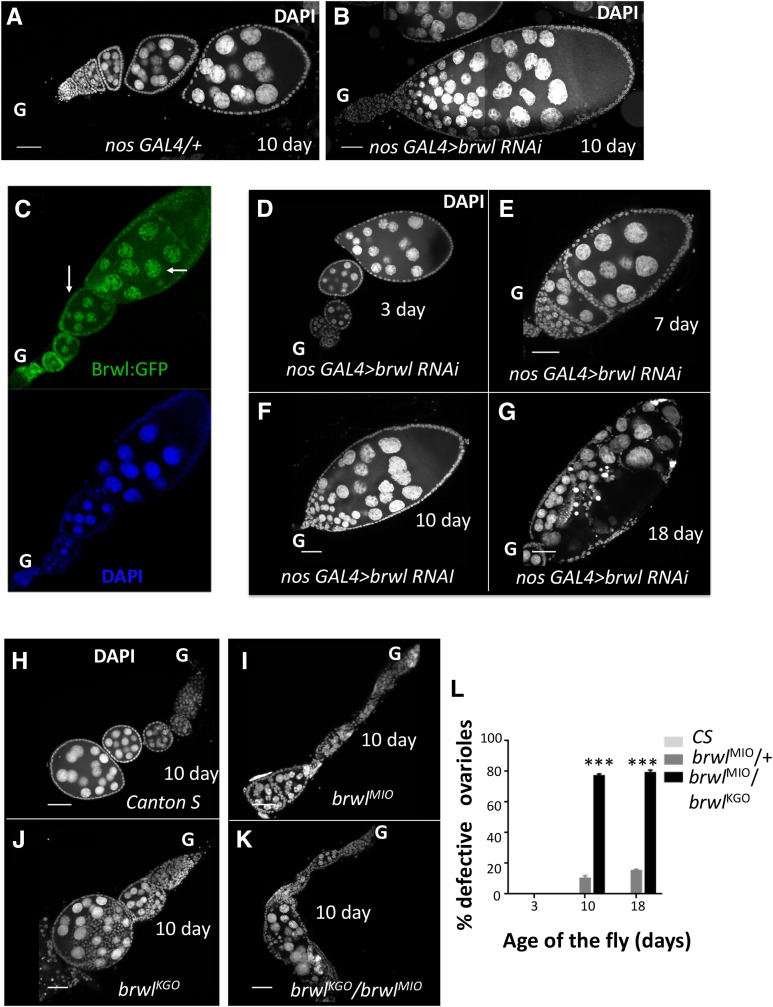
Compromising *CG3838/brwl* function in the female germline uncovers age dependent ovarian phenotypes. (A-B) Reduction of *brwl* transcripts in the female germline by expressing *brwl* TRiP VALIUM22 RNAi line, using *nos-Gal4* leads to sterility and a variety of ovarian defects in the adult female. Major morphological defects include fused ovarioles and pycnotic nuclei. (C) Brwl:GFP expresses in the nuclei of both germline and somatic cells. The staining resembles the expression of a Stwl protein trap line (Kelso *et al.* 2004) (Buszczak *et al.* 2007). The arrows indicate expression in nuclei of both germline (nurse cells) and the soma (follicle cells). (D-G) Ovarian defects are age dependent and manifested in the adult female 7-10 days (E-F) after of fly eclosion and the phenotypes are enhanced in older flies. 3 day old (D) animals show 100% normal oogenesis and lay fertile eggs while 18 day old (G) animals show ∼100% degenerated egg chambers. Nuclei are marked with DAPI. ‘G’ marks the anterior *i.e.*, germarial end of the egg chamber in all the figures. (H-K) Ovaries from females carrying *brwl* insertional alleles (*brwl^KG0^*, *brwl^MI0^*). The ovarioles are dissected from 10 day old females. Canton Special (Canton S) flies are used as controls. The oocytes show phenotypes similar to that of the *brwl* maternal RNAi knockdowns, with all ovarioles showing degenerating egg chambers by the 18^th^ day. (L) Quantitative data representing the percentage of ovarioles that show the phenotype on third, tenth (n = 80) and eighteenth (n = 25) day after fly eclosion. Ovaries from Canton-special (CS) animals were used as a control for statistical analyses. *, ** and *** denote *P* < 0.05, *P* < 0.01, and *P* < 0.001 respectively for this and all subsequent figures. N: number of adult females = 3-20 and n: number of ovarioles = 25-80.

In order to explore the possible germline specific function(s) of other MADF-BESS domain proteins, we sought to compromise the transcript levels of other MADF-BESS family genes in the female germline using a similar RNAi based approach. By employing the UAS-Gal4 system (Brand and Perrimon 1993; Duffy 2002), we tested MADF-BESS genes for which VALIUM20 (V20) and VALIUM22 (V22) RNAi lines (Hu *et al.* 2017; Perkins *et al.* 2015) are available (Table 1). *nos-Gal4* was employed as the primary Gal4 driver to knockdown the corresponding MADF-BESS genes specifically in the germline (Staller *et al.* 2013) by using both V20 and V22 lines. As summarized in Table 1 (column #3) compromising the function of only one other gene (CG3838), christened *brickwall* (*brwl*), resulted in ovarian phenotypes (Figure 1A-B). Interestingly, Yan *et al.* (2014) had earlier screened available VALIUM lines using both *nos-Gal4:VP16;UAS-Dicer2* and *Maternal Triple Driver (MTD)-Gal4*. Their studies, also summarized in Table 1 (fourth column), indicated that MADF-BESS genes, with the exception of *stwl* did not affect oogenesis significantly, after maternal knockdown of transcripts. The study did not uncover *CG3838* as a candidate possibly because *CG3838/brwl* mutant flies are fertile for the first ten days of egg laying, showing age-dependent loss of fertility, as described in the next section.

**Figure 2:**
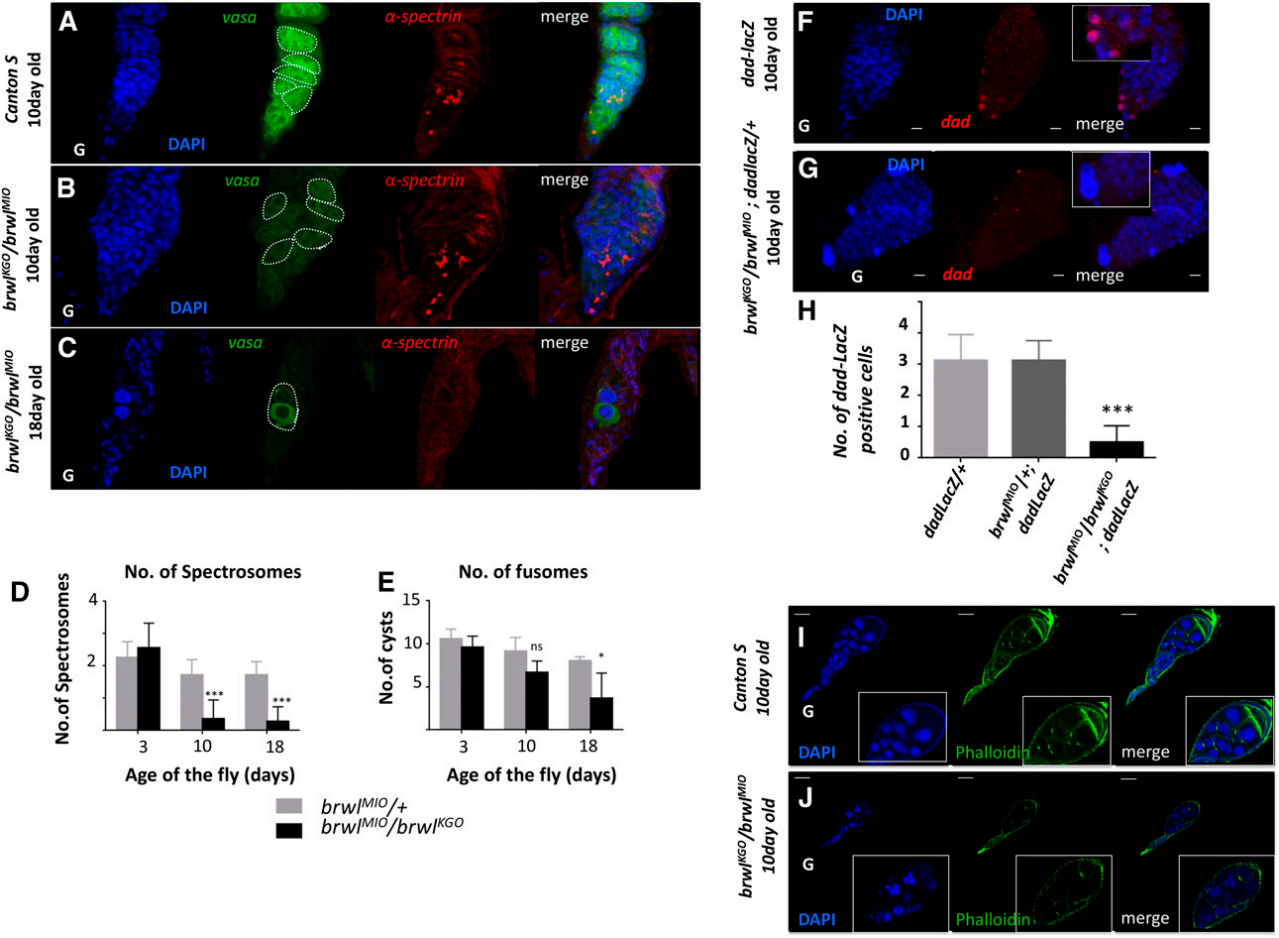
*brwl* alleles show defects in the cyst architecture and decrease in total number of germline stem cells with age. (A-C) *brwl^KG0^*/ *brwl^MI0^* mutants show decrease in Vasa expression (green) by the 10^th^ day PE (B) while α-spectrin staining (red) marks the number of spectrosomes, as measured at day 10 (B) and day 18 (C). N = 5, n = 15-35. Cysts are outlined with dashed lines. (D-E) Quantitative data representing number of spectrosomes (D) and fusomes (E) on third(n = 14), tenth(n = 22) and eighteenth day(n = 11) after fly eclosion. N = 5, n = 15-35. (F-H) *brwl^KG0^*/*brwl^MI0^* mutants show decrease in the number of *dad-LacZ* positive cells (G,H). LacZ levels, as measured by anti-LacZ antibody (red), is highest in germline stem cells of the ovary. N = 5, n = 15-30. Inset here and in following images is a 2X zoom of the main image. *dad-LacZ* line (F) is used as a control for this experiment. (I-J) *brwl^KG0^* / *brwl^MI0^* mutants show disorganization and decrease in number of ring canals as visualized by phalloidin staining (green). N = 5, n = 15-20.

The ovarian defects induced by the RNAi based knockdown of *brwl* prompted us to assess the expression of Brwl in the Ovary. Since antibodies against Brwl protein are unavailable, we used a Brwl:GFP fusion (BDSC 56150) line (See Materials and Methods) to monitor the expression of Brwl protein. Brwl:GFP is expressed in the adult ovarioles, where it marks the nuclei of both nurse and follicle cells in developing egg chambers, Stage-1 onwards (Figure 1C). This is similar to the expression of Stwl:GFP (See Figure 2H, Buszczak *et al.* 2007). We were unable to detect nuclear expression in the GSCs using the Brwl:GFP line. This was also true for the Stwl:GFP line in our hands and in published literature (Rohrbaugh *et al.* 2013). The evidence for nuclear GSC staining for Stwl is based on the use of an anti-Stwl antibody (Clark and McKearin 1996; Maines *et al.* 2007).

Unlike the *nos-Gal4* driver, MAT-Gal4 does not express in the GSCs (Perkins *et al.* 2015; Yan *et al.* 2014) and drives expression in the cystoblasts, Stage-1 onwards. MAT-Gal4; UAS-Brwl RNAi (V22) females (Suppl. Figure 1E) displayed similar age dependent phenotypes as in the case of *nos-Gal4*. Taken together, the RNAi based inactivation suggests that *brwl* activity is likely required in the germline from very early stages of development.

Interestingly, Brwl-GFP is also expressed in the somatic (follicular) nuclei (Figure 1C, Suppl. Figure 1B). This expression pattern raises the possibility that *brwl* function is also required in the soma for proper germline development. We tested this possibility by compromising *brwl* function in the somatic cells using a variety of somatic drivers, namely *GR1-Gal4*, *c587-Gal4*, *tj-gal4* and *hh-Gal4*. The VALIUM10 Brwl RNAi line (BDSC 31922) was used for these experiments. Surprisingly however, none of the resulting F1 females showed any significant ovarian phenotypes, even when the experiments were conducted at an elevated temperature (29°). To confirm these observations, we also tested *TJ-Gal4* in combination with *UAS-Dicer2* to enhance *brwl* knockdown and could not detect any role for *brwl* in the soma. Further, we tested all other MADF-BESS genes, using available V10 and V20 lines in combination with *c587-Gal4*. The lack of phenotypes (Table 1) suggest at best a relatively minor or insignificant role for *brwl*, as also for other MADF-BESS genes in the somatic cells of the ovary. Thus, while a role for *brwl* in the soma is possible, our experiments do not support this possibility.

### RNAi mediated germline knockdown of CG3838 uncovers age dependent ovarian phenotypes

To start characterizing the egg chambers compromised for *brwl* activity, the ovaries were first stained with DAPI, a DNA dye. Readily visible phenotypes upon *brwl* RNAi included defects in the ovariole architecture; fused egg chambers and pycnotic nuclei (Figure 1B). These defects were highly penetrant as substantial number of the ovarioles (50/69; 72%) displayed a combination of these phenotypes. As indicated in the previous section, the phenotypic consequences appeared to be specific as germline knockdown of other MADF-BESS genes did not display such phenotypes (Table 1) while somatic knockdown did not mimic the maternal phenotypes, when tested in flies 3, 10 and 18 days post-eclosion (PE).

Subsequent careful characterization revealed that the phenotypic consequences upon loss of *brwl* in the germline were age dependent. At 25 **°**C, females three days PE showed completely normal ovarian morphology (Figure 1D). Consistently, these females were fertile, and laid eggs that went through normal development post-fertilization (Figure 1D). By the 7^th^ day PE, the abnormalities in the ovarian structure became apparent with fused egg chambers (Figure 1E). This defect became even more penetrant and severe by the 10^th^ day PE (Figure 1F), where multiple instances of a single large ovariole *i.e.*, fused egg chamber with abnormally high number of nurse cells were observed. Moreover, the fused egg chambers showed clear signs of structural abnormalities and the presence of pycnotic nuclei. By the 18^th^ day PE (Figure 1G), all the ovarioles were partially or completely degenerated. As many of these phenotypes are reminiscent of *stwl* loss of function, *stwl* and *brwl* appear to be the only two members of the MADF-BESS family with functions in the GSC lineage during female oogenesis.

### Transposon insertion alleles of brwl display similar ovarian phenotypes as germline specific knockdown of brwl

To extend the RNAi dependent phenotypic analysis further, two independent transposon insertion lines in the *brwl* locus, referred to as *brwl^KG0^* and *brwl^MI0^* (See Materials and Methods) respectively, were tested for phenotypes during ovarian development. Both *brwl^KG0^* and *brwl^MI0^* females show similar range of ovarian defects when homozygous (Figure 1I, J). Notably, these defects are also age dependent and at day 10 PE, appear to be equally drastic as compared to those seen with the *brwl*-RNAi. *brwl^MI0^* is maintained over a balancer and very few homozygotes emerge while *brwl^KG0^* is a homozygous viable allele. Less than 5% of the total ovarioles display ovarian defects in older heterozygous *brwl^KG0^* /+ and *brwl^MI0^* /+ females. By contrast, significant structural deterioration and egg chamber fusions are observed in the ovaries derived from the *trans*-heterozygous *brwl^KG0^* / *brwl^MI0^* females (Figure 1K, L). Phenotypic similarities between the germline specific knockdown of *brwl* function and transposon insertion lines in the *brwl* locus further supported a germline specific requirement for *brwl*. We thus sought to analyze this activity further. Both the *brwl^KG0^*/*brwl^MI0^* and *brwl^MIO^*/*brwl^MI0^* were employed for subsequent characterization of the ovarian defects.

### brwl mutants show a range of ovarian defects

The phenotypic consequences due to loss of *brwl* are highly variable. Thus, to better understand possible function(s) of this novel player, we have undertaken a detailed characterization of the individual phenotypes associated with loss of *brwl* during different stages of ovarian development. As preliminary data (see above) suggested age dependent progression of the phenotypic severity, we analyzed individual phenotypic traits keeping in mind the age dependence.

*Drosophila* ovary is made up of 14-16 ovarioles that individually function as an egg production line. The germarium containing GSCs surrounded by the somatic cells is situated at the anterior tip of the ovariole. Typically, the GSCs divide asymmetrically to generate another stem cell and a daughter cell, which undergoes differentiation. Stem cell daughters, or cystoblasts, undergo four rounds of synchronous division to eventually give rise to 16 interconnected cystocytes. While niche derived non-autonomous signaling coordinates GSC specification, cell intrinsic mechanisms contribute to the regulation of both the symmetric as well as asymmetric germline stem cell divisions. The unequal nature of division is reflected in the characteristic morphology of fusomes which are the membranous organelles rich in proteins such as α-Spectrin and, Hu-li tai shao (Hts) (*Drosophila* homolog of Adducin). Thus, GSCs can be readily identified by the virtue of the presence of a round fusome also referred to as spectrosome, located adjacent to the somatic cap cells. By contrast, the differentiated progeny of GSCs *i.e.*, cystocytes or cystoblasts display diagnostic changes in the shape of the fusome including elongation and bifurcation. Since *stwl* function is required in the GSCs for their maintenance, we first decided to assess if *brwl* mutants also show a GSC specific phenotype.

*brwl mutants affect cyst architecture and GSC number in the early stages of oogenesis:* Preliminary analysis of loss of function mutants in *brwl* locus as well as germ line specific knock down of *brwl* function indicated involvement of *brwl* during several stages of ovarian development. To better understand the function of *brwl* in the process we decided to take a closer look at the germaria from the *brwl* mutant females. To this end *brwl^KG0^*/*brwl^MI0^* ovaries were first stained with anti-Vasa antibodies and counterstained with DAPI. *Vasa* is a maternal effect, posterior group gene and *vasa* RNA as well as Vasa protein are localized in the pole plasm that is assembled and subsequently anchored at the posterior pole of the oocyte. Vasa protein is enriched in a germline specific manner through the course of development. Accordingly, within early germarium Vasa protein is present in germ cells including GSCs and thus has served as a reliable marker of the germline fate (Lasko and Ashburner 1990). In ten day-old *brwl^KG0^*/ *brwl^MI0^* ovaries, Vasa protein appears to be considerably less than the wild type (Figure 2B). Additionally, the staining pattern also revealed that Vasa positive cysts appear disorganized and are distributed randomly within the germarium (Figure 2B). By the 18^th^ day, as the number of cysts degenerate, *brwl^KG0^*/*brwl^MI0^* ovaries show presence of considerably larger than average sized nuclei (Figure 2C) possibly due to apoptosis (see below). Presence of Vasa around these nuclei also suggests that such cysts might have division defects.

To specifically analyze the influence of Brwl on the GSCs and their progeny, we stained the *brwl^KG0^*/*brwl^MI0^* ovaries with antibodies against α-Spectrin and counted the number of spectrosomes and fusomes (Figure 2D-E). Typically, wild type germarium consists of 2-3 GSCs, which can be readily identified by the presence of spectrosome. By contrast, in the ovaries derived from 10 days old *brwl^KG0^*/*brwl^MI0^* females, total number of cells with a spectrosome were substantially reduced. At the 18^th^ day, in several germaria, not even a single cell positive for α-Spectrin could be detected (Figure 2C, D), indicating a substantial decrease in the total number of GSCs and cystoblasts. A number of fusomes are present in the wild type is between 9-11. Interestingly in the ovaries from the *brwl^KG0^*/*brwl^MI0^* 10-day old post-eclosion females, no significant decrease in the number of fusomes was observed. However, this number decreased substantially in the 18^th^ day old flies (3-5 fusomes) (Figure 2E). Moreover, unlike the wild type female ovary samples, *brwl^KG0^*/ *brwl^MI0^* ovaries also exhibited disorganized cysts that have lost a stereotypical linear arrangement. Instead of canonical 2, 4, 8, and 16 cell cyst organization, they were unevenly distributed in the mutant germarium.

*brwl mutants show decrease in the number of GSCs*: In wild type females, total number of GSCs decreases with age. A single ovariole generally consists of 2-4 GSCs which are maintained until late in the lifespan and the GSC count decreases by half only by day 63 post-eclosion (Casanueva and Ferguson 2004; Pan *et al.* 2007). *brwl^MI0^*/ *brwl^MI0^* ovaries show considerable decrease in the number of spectrosome positive cells (Figure 2G,H) between day 10 through 18 post-eclosion. This observation taken together with disorganized cysts with reduced levels of Vasa protein indicated that *brwl* activity is likely needed for GSC specification and/or maintenance. To confirm this directly a GSC marker *dad (daughters against dpp)-lacZ* was used to stain the GSCs. *brwl^KG0^*/ *brwl^MI0^* ovaries show a 60% reduction (Figure 2H) in the *dad-lacZ* positive cells 10 days after eclosion.

In a wild type germarium, a cyst consists of 16 cells that are interconnected via cytoplasmic bridges also known as ‘ring canals’. As the egg chambers derived from *brwl^KG0^*/ *brwl^MI0^* adult female ovaries appeared to display variety of structural defects, we decided to characterize the cytoskeletal defects by analyzing ring canals, which are actin rich structures. We stained the *brwl* mutant ovaries with fluorescently labeled phalloidin (Figure 2J**)**, which labels actin. *brwl^KG0^*/ *brwl^MI0^* ovaries show significant loss of ring canals (∼70% reduction as compared to Cantos S), critical for oocyte development from day 10 onwards. Fusome, a branched structure made up of continuous endoplasmic reticulum and cytoskeletal components is responsible in determining the mitotic division plane. Because of this influence, eventually after 4 mitotic cell divisions, only two cells end up with four ring canals. One of these two cells eventually would assume the oocyte identity while forcing the other to acquire a nurse cells fate like the remaining cells with fewer number of ring canals. Nurse cells are responsible for the production of cytoplasmic components such as proteins and RNA that are trafficked to the oocyte. As ring canals are the structures through which nurse cells pass the nutrients to the oocyte, reduced number of ring canals raised obvious questions about the oocyte identity in *brwl^KG0^*/ *brwl^MI0^* egg chambers.

**Figure 3:**
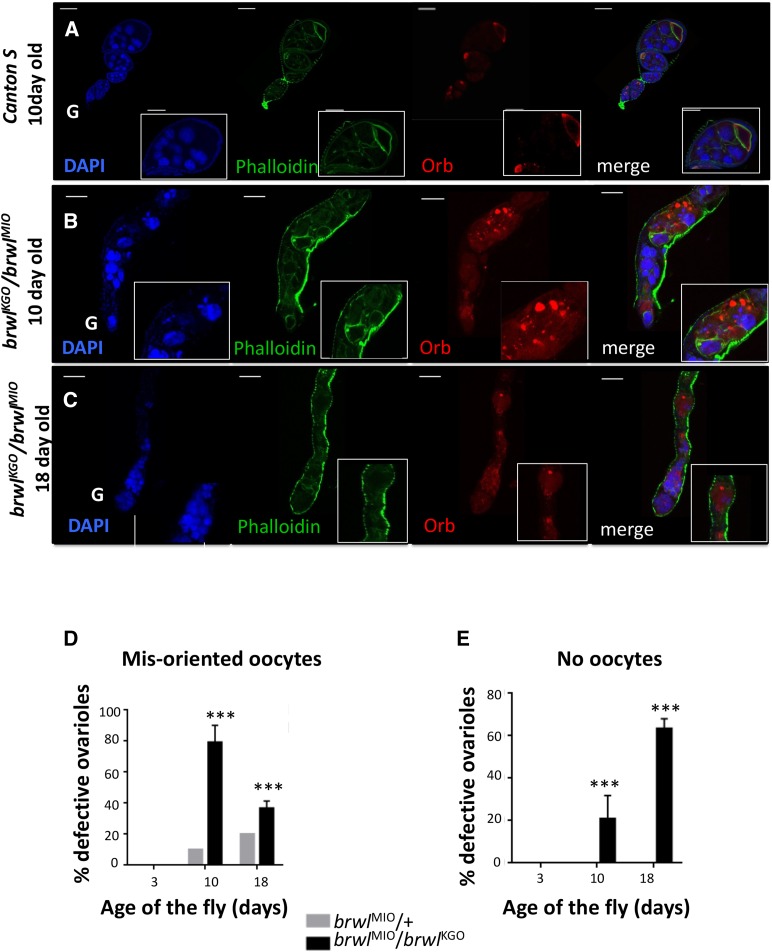
Orb is mislocalized or absent in *brwl* mutants. (A-C) *brwl^KG0^*/ *brwl^MI0^* mutants display defective ovarioles. These defects were classified in two major categories, mis-positioned oocytes and no-oocytes, based on actin (green) and Orb (red) staining. Orb related phenotypes enhance with age, with 18 day old flies having a high proportion of ovarioles that have fewer (C) or no oocytes (Suppl. Figure 1). DAPI (blue), F-actin (green) and Orb (red) are used to mark ovarioles. Insets are a 2X Zoom of the images. (D-E) Quantitative data representing the percentage of ovarioles that show the phenotype on third, tenth and eighteenth day after fly eclosion. Ovaries from Canton S 10 day old *brwl^MIO^/brwl^KGO^* animals were used as a control for statistical analyses. N = 3-15, n = 30-50.

*brwl mutants affect oocyte specification:* Proteins like Orb, Egl and BicD (Huynh and St Johnston 2000) that accumulate in the prospective oocyte are essential for oocyte specification and axis determination. *orb* encodes for *Drosophila* homolog of **C**ytoplasmic **P**olyadenylation **E**lement **B**inding protein, (CPEB) and has been shown to be required at multiple levels during oogenesis including formation of a 16-cell cyst, oocyte specification, and polarity establishment and/or maintenance (Lantz *et al.* 1994). Orb accumulation is progressively restricted to the presumptive oocyte during early stages of oogenesis. Furthermore, Orb is localized in a crescent shaped pattern at the posterior end of the developing oocyte. To assess if loss of *brwl* influences oocyte specification, *brwl^KG0^*/ *brwl^MI0^* ovaries were stained with anti-Orb antibodies (Figure 3A-C). The staining pattern of Orb protein in mutant egg chambers suggested age dependent loss of oocyte specification. Other phenotypes included mis-positioned oocytes or complete loss of oocyte identity. On several occasions although present, Orb protein was not restricted to a single cell and was instead detected in a group of cells. Consequently, by 10^th^ day, 77.6% of the total ovarioles (n = 45) showed either mis-positioned oocyte and/or several mis-specified *i.e.*, Orb positive cells (Figure 3B). Moreover, 20.83% egg chambers showed absence of a presumptive oocyte as assessed by lack of Orb accumulation in any cell (Suppl. Figure 1D). By the 18^th^ day 63.3% of the ovarioles (n = 32) showed egg chambers with a complete absence of oocyte, while around 36.7% showed mis-positioned oocytes (Figure 3D,E). This demonstrates progressive worsening of the phenotype and deterioration of the tissue from day 10 to day 18.

**Figure 4:**
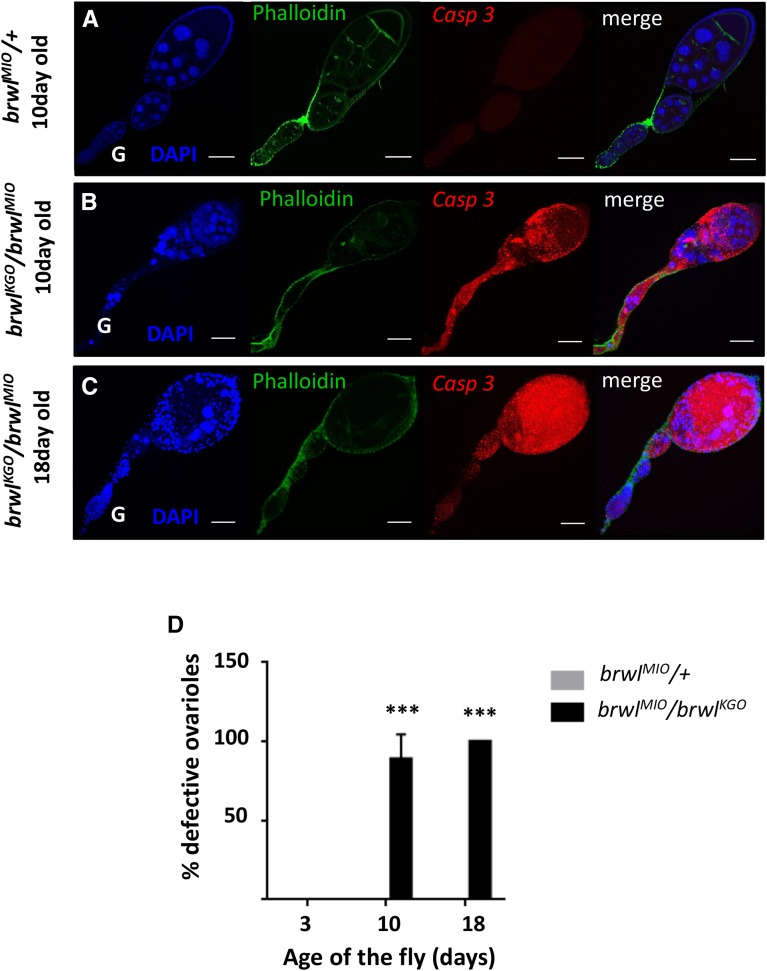
Caspase is activated in *brwl* mutants. (A-C) *brwl^KG0^*/*brwl^MI0^* mutants are positive for apoptosis as stained by apoptotic marker cleaved caspase-3 (Casp 3). The intensity of staining is enhanced as the fly grows older. 18 day old animals (C) show 100% ovarioles with activated caspase. DAPI (blue), Phalloidin (green) and activated caspase-3 (red) stained ovarioles. (D) Quantitative data representing the percentage of ovarioles that show the Casp 3 staining on third, tenth and eighteenth day after the fly eclosion. Ovaries from three day old *brwl^MIO^/brwl^KGO^* animals were used as a control for statistical analyses. N = 3-15, n = 15-40.

*brwl mutants show age dependent Caspase activation:* DAPI staining of 10 day *brwl*^KG0^/*brwl^MI0^* mutants showed pycnotic nuclei indicative of apoptotic cell death. To test whether this is indeed the case, *brwl*^KG0^/*brwl^MI0^* ovaries were stained with cleaved Caspase-3 antibody (Sarkissian *et al.* 2014), which is a marker for cells undergoing apoptosis. *brwl* mutant ovaries undergo cell death as shown (Figure 4A-D). 88.88% (n = 39) of ovarioles show extensive apoptosis as assessed by the presence of cleaved Caspase on the 10^th^ day after fly eclosion which deteriorates further by 18^th^ day post-eclosion as all the ovarioles positive for caspase activity (n = 28). This drastic increase in cell death likely results in oogenesis arrest in these mutants (Buszczak and Cooley 2000).

**Figure 5:**
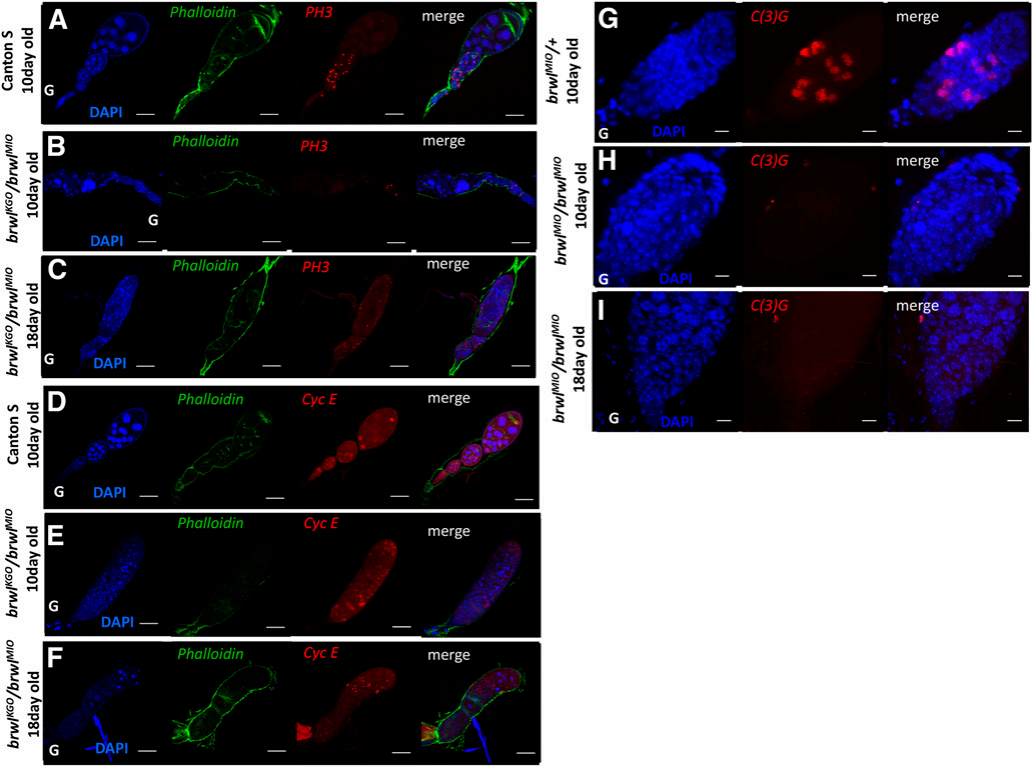
*brwl* mutants affect mitotis and meiosis. (A-C) *brwl^KG0^*/*brwl^MI0^* mutants show decrease in the number of mitotically active cells, as stained by a phospho-histone3 S10 (PH3) antibody (red) and counterstained by phalloidin (green), in 10 day old ovaries. This pattern appears abrupt and random by 18^th^ day where mitotic cells are seen randomly situated in the ovariole. N = 5-7, n = 20 = 25. (D-F) *brwl^KG0^* /*brwl^MI0^* mutants show decrease in the number of mitotically active cells, as stained by the Cyclin E antibody (red) in 10 day old ovaries. This pattern is further disrupted by 18^th^ day (C) where randomly scattered mitotic cells are seen in the ovariole. N = 5, n = 15-35. (G-I) *brwl^MI0^*/*brwl^MI0^* mutants show complete loss of the formation of synaptonemal complex, as marked by the anti-C(3)G antibody, on the 10^th^ day post eclosion. 100% of ovarioles show this phenotype. N = 5, n = 15-25.

*Mitosis and Meiosis is affected in brwl mutants*. Mitosis in *Drosophila* ovaries is tightly regulated at multiple levels by different pathways (Deng *et al.* 2001). Wild type ovaries when stained for phospho-histone-3 S10 (PH3) show mitotically active cells until stage 6 (Figure 5A-C) after which they enter an endoreplication phase. Interestingly, in *brwl*^KG0^/*brwl^MI0^* ovaries this pattern of mitosis is lost. It is difficult to stage the mutant ovary samples owing to degeneration, but it appears that the total number of mitotic nuclei is considerably reduced in *brwl* egg chambers. In 10 days old ovaries very few, and widely distributed mitotically active cells are observed (Figure 5B) whereas by 18^th^ day old (Figure 5C) unevenly distributed mitotically active cells are seen throughout the ovariole. We confirmed this by staining the ovaries with anti-cyclin E antibody, which also shows an aberrant pattern (Figure 5E-F) and is indicative of cell division defects in *brwl*^KG0^/*brwl^MI0^* ovaries.

Meiosis initiated in the cysts after 4^th^ mitotic division is essential for oocyte specification in *Drosophila* ovary. It has been shown that onset of meiosis occurs in 2 pro-oocytes with 4 prospective ring canals after the meiotic markers accumulate in these 2 cells (Carpenter 1975) (Jeffress *et al.* 2007; Lake and Hawley 2012). Failure of proper cyst formation and accumulation of meiotic markers hinders oocyte specification (Huynh and St Johnston 2000). Since *brwl* mutants showed defects in oocyte specification we stained the *brwl* mutants with a meiosis marker C(3)G which marks the synaptonemal complex in a meiotic cell (Jeffress *et al.* 2007). *brwl* mutants show complete loss C(3)G staining on 10^th^ day post eclosion (Figure 5G-I). This indicates that none of the cells in cysts form the synaptonemal complex. This is similar to that of BicD null mutants where oocyte specification is not achieved (Huynh and St Johnston 2000).

### brwl mutant phenotype is rescued by over-expression of stwl in germ stem cells

We are interested in the sequence homology and its relationship with the functional redundancy among the members of MADF-BESS family of proteins. Detailed phenotypic characterization of *brwl* mutants revealed remarkable overlap with *stwl* loss of function. Both the proteins seem to influence similar phenotypic traits including the establishment and/or maintenance of GSC fate, proper cystoblast differentiation and oocyte specification. We thus wondered if the two proteins function in a redundant manner. To directly test this possibility, we sought to assess if *brwl* mutant phenotypes could be rescued by expression of *stwl*. As a control, we also decided to test other MADF-BESS domain proteins in an identical experimental set-up. Among the different lines tested *UAS-CG13204*, *UAS-CG3838*, *UAS-stwl*, *UAS-CG4404*, *UAS-CG8359*, *UAS-Coop* and *UAS-CG3919* showed expression in the ovary, when driven by *nos-Gal4* and as monitored by HA staining. Unfortunately however, flies of only two genotypes survived to adulthood and could be analyzed further: *brwl^MIO^/Brwl^KGO^*; *nos-Gal4/UAS-stwl* and *brwl^MIO^/brwl^KGO^*; *nos-Gal4/UAS-CG3919*. All the other genetic combinations failed to eclose making further analysis untenable.

**Figure 6:**
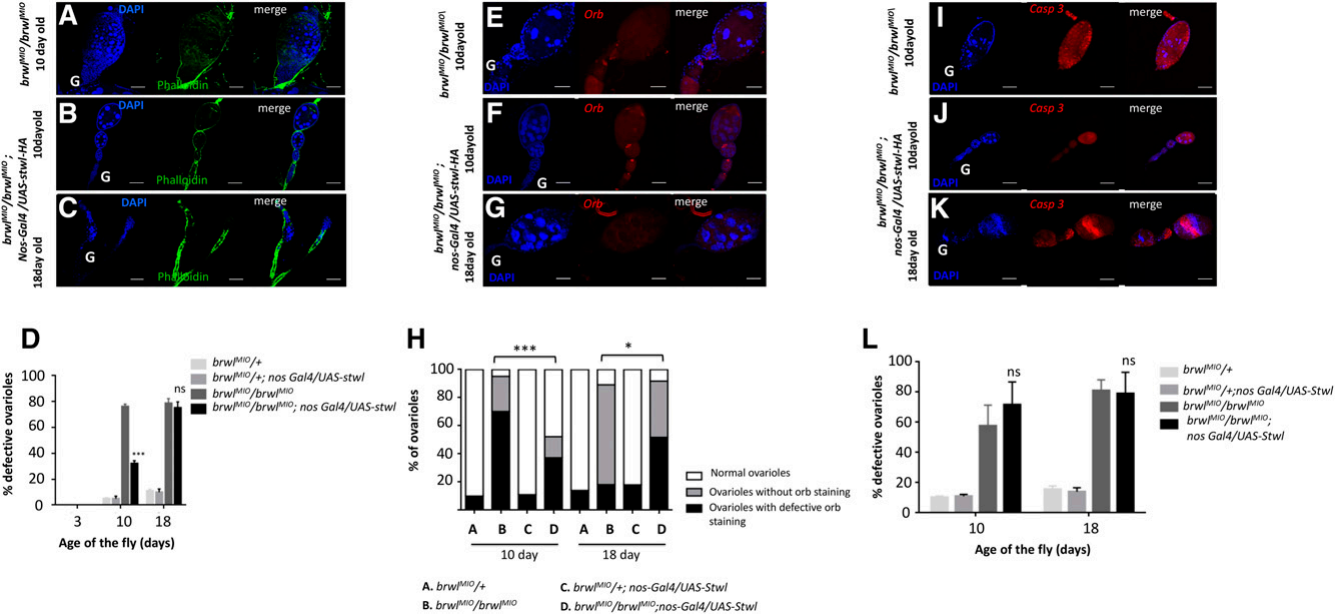
Ovarian defects due to loss of *brwl* are ameliorated by over-expression of *stwl*. (A-C) *brwl^MI0^*/*brwl^MI0^* mutants show ovary defects, as measured by DAPI staining (blue) and phalloidin (green) that are rescued in *brwl^MI0^*/ *brwl^MI0^*; *nos-Gal4/UAS-stwl-HA* on the 10^th^ (B) and 18^th^ (C) day PE. The rescue is however not as strong on the 18^th^ day PE. (D) Quantitative data representing the number of ovarioles showing the phenotype on 3^rd^ (n = 10), 10^th^ (n = 56) and 18^th^ (n = 25) day after fly eclosion. Ovaries from three day old *brwl^MIO^/brwl^KGO^* animals were used as a control for statistical analyses. (E-G) *brwl^MI0^*/*brwl^MI0^*; *nosGal4/UAS-stwl-HA* ovarioles showing reduced oocyte specification defects. Oocyte defects are significantly rescued in *brwl^MI0^*/ *brwl^MI0^*; *nosGal4/ UAS-stwl-HA* ovarioles on the 10^th^ day (n = 25). Mild rescue is seen on the 18^th^ day (n = 15) with a decrease in ovarioles without orb staining. (H) Quantitative data representing the percentage of ovarioles that show the phenotype on third, tenth and eighteenth day after fly eclosion. Ovaries from three day old *brwl^MIO^/brwl^KGO^* animals were used as a control for statistical analyses. N = 3-15; n = 25-60. (I-K) *brwl^MI0^*/ *brwl^MI0^* mutants show apoptotic egg chambers which is not rescued in 10 day old *brwl^MI0^*/ *brwl^MI0^*; *nanosGal4/ UAS-stwl-HA* ovarioles. (L) Quantitative data representing the percentage of ovarioles that show the phenotype on third, tenth (n = 20) and eighteenth (n = 15) day after fly eclosion. Ovaries from three day old *brwl^MIO^/brwl^KGO^* animals were used as a control for statistical analyses. N = 3-10; n = 15-20.

Interestingly we observed a substantial rescue of several phenotypes associated with loss of *brwl* upon germline specific *stwl* expression whereas CG3919 expression was unable to achieve rescue. Overexpression of *stwl* with *nos-Gal4* in *brwl^MI0^*/ *brwl^MI0^* background led to an amelioration of the ovarian phenotype induced by loss of *brwl*. For instance, ovarioles from 10 day old female ovaries consisted of normal egg chambers with 15 nurse cells and a properly developing oocyte (Figure 6A-B, E-F). Only 32.8% of total *brwl^MI0^/brwl^MI0^*; *nanos-Gal4/UAS-stwl-HA* ovarioles (n = 56) show ovarian defects which is substantially lower as compared to *brwl^MI0^*/ *brwl^MI0^* ovarioles. Moreover, considerable morphological improvement was also observed as *brwl^MI0^/ brwl^MI0^*; *nos-Gal4/UAS-stwl-HA* ovarioles display regularly spaced ring canals with in the egg chambers (Figure 6B). Notably however, overexpression of *stwl* is unable to sustain the rescue levels for the 18-day old ovarioles of *brwl* mutant females (n = 25) (Figure 6C).

Overexpression of *stwl* in *brwl^MI0^*/ *brwl^MI0^* animals can also partially rescue the oocyte specification defects as assessed by Orb staining (Figure 6F,H). In *brwl* mutants the percentage of ovarioles with defective oocytes (no oocytes + mis-patterned oocytes) on the 10^th^ day is ∼95%, with <5% normal oocytes. Expression of *stwl* in *brwl^MI0^*/ *brwl^MI0^* leads to a dramatic rescue with >50% of the ovarioles (n = 25) showing normal patterning as compared to <5% in the mutant. The rescue at day 18 is also significant (Figure 6G,H) with egg chambers without Orb staining decreasing from 80% (in *brwl^MI0^*/ *brwl^MI0^)* to 40% after Stwl overexpression. This clearly indicates that overexpression of *stwl* in *brwl* mutants delays the accumulation of *brwl* associated defects.

Lastly, we examined if the germ cell division pattern is restored by overexpression of *stwl* in *brwl* loss of function background. As judged by anti-pH 3 S10 (Suppl. Figure 2A) and anti-CyclinE (Suppl. Figure 2B) stainings the 10-day old *brwl^MI0^/ brwl^MI0^*; *nanos-Gal4/UAS-stwl-HA* ovarioles show marked improvement. However, consistent with earlier observations mitotic defects persist in the 18^th^ day *brwl^MI0^/ brwl^MI0^*; *nanos-Gal4/UAS-stwl-HA* ovarioles. Taken together these data argue that *bwl* activity is continuously required during germline development and Stwl protein can partially substitute loss of *brwl* albeit in a temporally restricted manner.

Consistent with this limited ability to rescue overexpression of *stwl* in *brwl^MI0^* / *brwl^MI0^* animals did not decrease the apoptotic cell death that *brwl^MI0^*/ *brwl^MI0^* ovarioles show on 10^th^ (n = 20) and 18^th^ day respectively (n = 15) (Figure 6I-L). The temporal rescue of Brwl by Stwl does not appear to extend to blocking the apoptotic program that gets initiated in the absence of *brwl* function. It is however possible that higher dose of Stwl protein may be sufficient to rescue all the phenotypes due to loss of *brwl* effectively and additional experiments involving better reagents will be required to examine this possibility.

### Negative purifying selection for stwl and brwl

**Figure 7:**
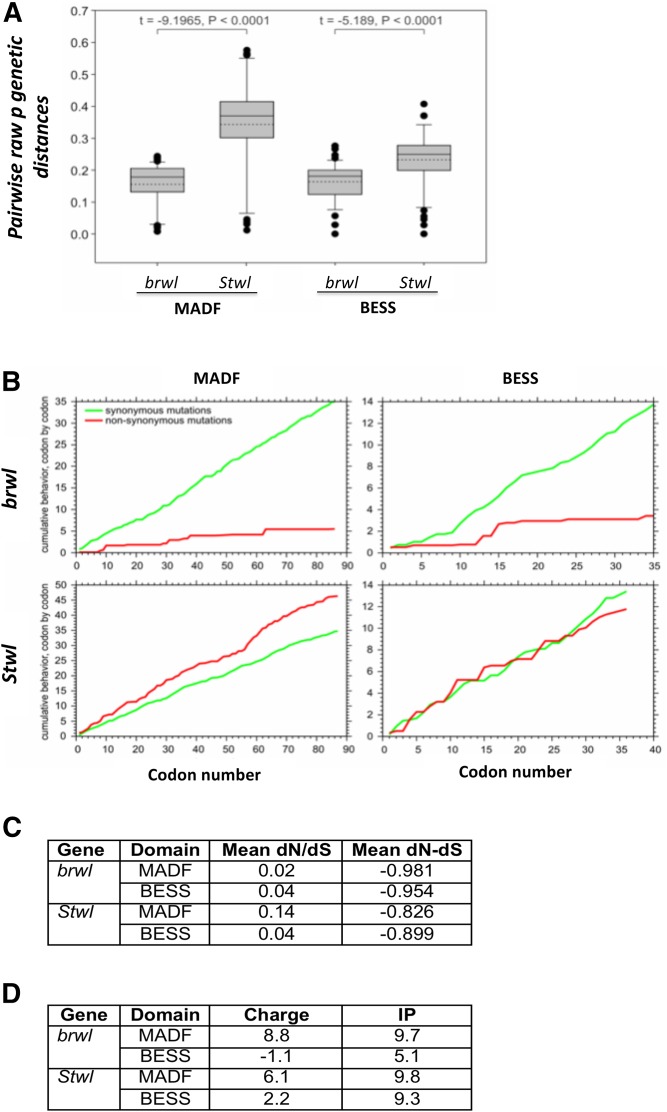
Evolutionary analyses of changes in nucleotide and amino acid sequences in the MADF-BESS family. (A) Box plot for divergence of nucleotide sequences for *stwl* and *brwl* for 12 *Drosophila* genomes. Solid line in the box is median, dashed line is mean and outliers are shown by solid circles. (B) Cumulative behavior of synonymous and non-synomous substitutions along the coding region of *stwl* and *brwl*, showing accumulation of amino-acid substitutions within the MADF or BESS domains for 12 *Drosophila* genomes. The X-axis represents the position of the codon, and the Y-axis represents the average cumulative number of synonymous (green line) and non-synonymous (red line) codons, estimated at a specific codon position. (C) dN/dS ratio as also mean dN-dS for pairwise comparison between species computed for MADF and BESS coding regions of 12 *Drosophila* genomes. (D) Predicted charge and Isoelectric point (IP) of MADF and BESS domains in Stwl and Brwl. The values are compared with published values for MADF domain containing proteins (Maheshwari *et al.* 2008).

Taken together our analysis of Brwl and Stwl suggests that these two proteins are similar enough but they may have retained independent functions. We decided to explore this possibility using phylogenetic analysis. In an earlier study (Shukla *et al.* 2014), we found evidence for gene duplication and lineage specific expansion of the entire MADF-BESS family. Since our experimental data suggests that *stwl* and *brwl* are functional duplicates in the MADF-BESS family, we analyzed codon substitution patterns for the family with a specific focus on *stwl* and *brwl* (See Materials & Methods). We find that the mean pairwise raw genetic distance was two times higher in MADF of *stwl* than *brwl* and 1.4 times higher in BESS of *stwl* than *brwl* (Figure 7A). This indicates that the rate of evolution of both MADF and BESS domains of *stwl* was significantly higher than that for *brwl*. Model selection for nucleotide substitutions suggests that the first two codon positions are evolving by either simple one parameter (Jukes and Cantor 1969) model with equal rates for substitutions of the four nucleotides or with a two parameter (Kimura 1980) model with different rate of transition and tranversion type of nucleotide substitutions (Suppl. Table 1). Third codon position showed a complex model of nucleotide substitution only for MADF domain of *brwl*, while other domains showed simple substitutions with two parameters (Suppl. Table 1). A cumulative dN and dS plot (Figure 7B) indicates that MADF and BESS domains of *stwl* accumulated higher number of non-synonymous substitutions as compared to the respective domains of *brwl*, probably because of their higher rate of mutation. This suggests that *brwl* is under more stringent-evolutionary control in terms of codon substitutions than *stwl*. The dN/dS ratio for pairwise comparison between species showed that for both MADF and BESS domains of *stwl* and *brwl* was much less than one (Figure 7C), indicating that there is purifying or negative selection. This was also evident from negative dN-dS values for codon-by-codon analysis considering best fit codon substitution model (Figure 7C). Negative or purifying selection indicates selective removal of deleterious alleles, suggesting vital role of the domains and by association for both Brwl and Stwl in cellular functions. Stwl and Brwl have thus maintained their amino acid sequences in spite of changes to the DNA coding sequences, over time. The above analysis thus supports that Brwl and Stwl perform overlapping but distinct functions.

(Maheshwari *et al.* 2008) analyzed the charge and isoelectric point (IP) based on amino-acid sequence of MADF domains in *Drosophila* and discovered that proteins with multiple MADF domains in a single sequence, for example hybrid male resce (Hmr) and lethal hybrid rescue (Lhr) have distinct Charge and Isoelectric points. They reasoned that MADF domains with a strong positive charge were DNA binding proteins and those with negative charge were primarily binding basic proteins like histones. We find that Stwl and Brwl, having positive Charge of 6.1 and 8.8 respectively (Figure 7D) for their respective MADF domains fall in the category of transcription factors of the type ‘MADF1’, geared for DNA binding (Compare to Table1, (Maheshwari *et al.* 2008)). In contrast, the BESS domain appears to differ significantly in its charge and IP for Brwl as compared to Stwl, suggesting a possible difference in interactors between the two proteins.

## Discussion

The continuity of a species is almost exclusively dependent upon the transmission of genetic information across generations. Multicellular organisms accomplish this task via sexual reproduction, which typically involves fusion of the male and female gametes resulting in the development of a new organism post-fertilization. The progenitors of the male and female gametes, known as GSCs, are thus under constant scrutiny. Consequently, a wealth of information is available regarding the mechanisms underlying the specification and maintenance of the GSC fate. How the specification process follows a defined route to eventually accomplish a reliable outcome namely continuous production of healthy gametes is less clear, however. This is of special significance as GSCs not only self-renew but are also pluripotent and are competent to give rise to daughter progeny that subsequently differentiates into a variety of cell types. Thus, it is important to elucidate how GSCs maintain ‘stemness’ by employing symmetric cell division and also have the capability to generate differentiated daughter cells via asymmetric cell division program.

It is proposed that the process of GSC specification relies upon mechanisms that would ensure both qualitatively and quantitatively robust outcomes (Rudel and Sommer 2003; Félix and Barkoulas 2015; Kitano 2004). To achieve dependable and reproducible outcomes, developmental processes are often reinforced by redundant mechanisms that effectively allow dampening of potential disruptions in the functional gene regulatory network. ‘Gene duplication and sub-functionalization’ is a prominent strategy among the different safeguarding means that have been employed to generate redundancy (Gu 2003; Kitano 2004). Duplicated genes ensure robustness in a system as deleterious mutations on one gene are readily compensated by the activity of the duplicated member. Gene duplicates thus provide functional redundancy, buffering detrimental phenotypes that arise from malfunction of one copy (Gout and Lynch 2015; Shukla *et al.* 2014). This contributes significantly to the developmental transitions occurring in a sequential and seamless manner from one stage to the next.

In this study, we have presented experimental analysis of two paralogs *stwl* and *brwl*, which appear to be partially redundant molecular players that perform similar functions and thus contribute to the robustness to the processes underlying GSC development in *Drosophila* ovaries. *brwl* appears to function in the same cell lineage and spatiotemporal domain as *stwl*, and *stwl* expression in the germ cell lineage *i.e.*, *nos* domain can partially rescue/delay *brwl* phenotypes. Our study finds that similar to *stwl*, *brwl* is also required for stem cell maintenance, proper cyst formation and oocyte specification.

First, we examine the implications of the evolutionary data analyzed. Gene duplicates are believed to evolve differently than singletons (Jordan *et al.* 2004). Post-duplication, the genes could show changes in their rates of evolution with consequences on purifying (negative or positive) selection. Our data, based on the analysis of the MADF and BESS domains suggest that a) *stwl* and *brwl* have undergone negative selection, indicating that their ancestral functional state, as defined by the linear sequence of amino acids was critical and had to be maintained and that b) The rate of evolution appears asymmetric with MADF and BESS domains coding regions of *stwl* and *Brwl* sequences changing at different rates.

We have compared the *stwl* phenotypes described in literature with our data as we were unable to collect data for a direct comparison due to unavailability of published Stwl reagents (Clark and McKearin 1996; Akiyama 2002), such as mutant alleles and antibodies. *brwl* loss of function shows strong age dependent phenotypes that were not reported in the analysis of any *stwl* mutants. Both *stwl* and *brwl* show a range of similar phenotypes, specific to the female germline. The ability of *stwl* to partially compensate for *brwl* function indicates that *stwl* may be a genetic interactor of *brwl* and that Stwl has function similar to Brwl and can replace, at least partially, Brwl, suggesting redundancy in function. Since both proteins are MADF-BESS domain proteins and predicted paralogs, this leads to a strong possibility that the common function shared by both is an ancestral function retained by both orthologs.

**Figure 8:**
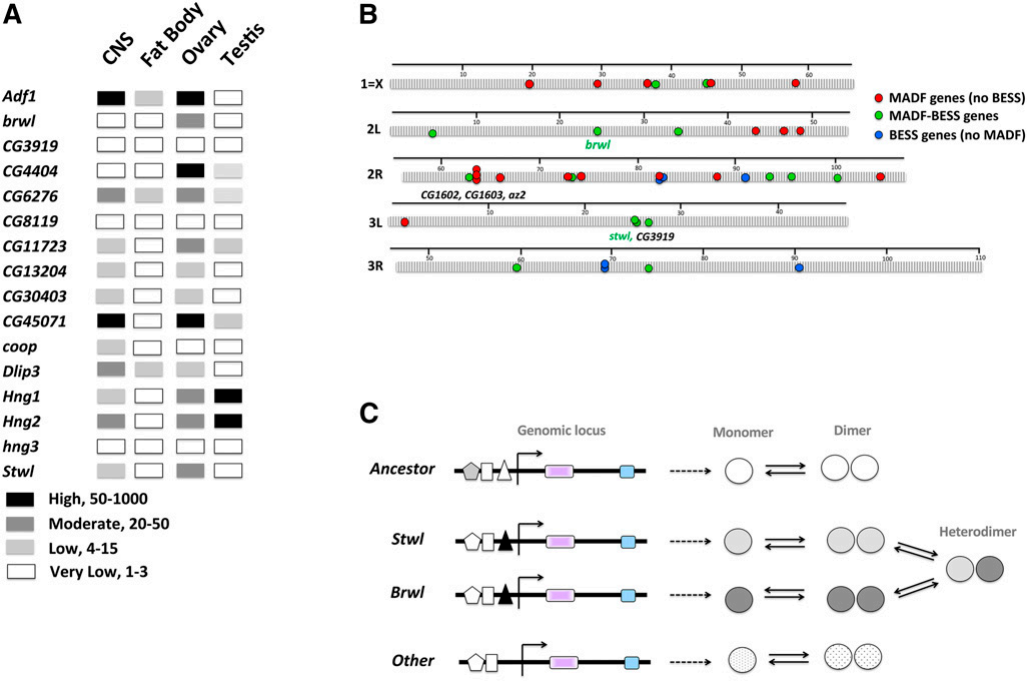
A model for Stwl/Brwl function post-duplication and after subsequent Sub-functionalization. (A) MADF-BESS mRNA expression, extrapolated from modENCODE datasets in larval CNS, larval fat body, adult ovary and adult testis of *Drosophila melanogaster*. These examples highlight the varied expression pattern of these genes indicating that the family members are sub-functionalized in terms of spatiotemporal expression pattern. The levels of transcripts (High, Moderate, Low) are based directly on values from modENCODE. (B) Schematic of genomic organization of MADF-BESS loci. MADF (red circles), MADF-BESS (green circles) and BESS (without MADF, blue circles) domain coding genes are distributed all over the genome. *stwl* and *brwl* are on different chromosomes and *CG3919*, a local duplicate of *stwl* does not appear to have functional roles in the GSC lineage. The map distances are in centimorgans and genetic distances are based on chromosomal maps, as collated by Flybase. (C) *brwl* and *stwl* are expressed in overlapping domains (driven by enhancer, filled triangle) in the GLC lineage and we hypothesize that the functional entity for this overlapping function, which is critical for robust function, is a Stwl/Brwl heterodimer and absence of the Brwl homodimer can be functionally replaced, to a limited extent by a Stwl Homo-dimer.

This thesis is further supported by available evidence as summarized below. A comparison of modENCODE data indicates that both *brwl* and *stwl* are expressed in the early embryo likely due to maternal deposition. Transcript levels of both the genes are higher in the ovaries as compared to testis (Figure 8A) consistent with the elevated levels in adult females. Some MADF-BESS genes (*Hng1*, *Hng2*) show similar expression patterns with others showing varying levels of expression in different tissues. In contrast to the data that shows equivalence for Brwl and Stwl function, there is also evidence for differences between the two indicating gain/loss of function for one member. For example, Loss of *stwl* in the wing by RNAi shows wing/wing-hinge phenotypes whereas *brwl* RNAi has no effect (Shukla *et al.* 2014). Overexpression of *stwl* perturbs development, both in wings and in the ovarian stem cell lineage. *brwl* overexpression on the other hand has no such effect. Loss of *brwl* leads to a decrease in GSC number, with survivability being one of the possible means by which Brwl regulates GSC number.

A genomic map of distribution of MADF-BESS genes reveals interesting features (Figure 8B). The MADF-BESS genes (green circles) are distributed all over the genome, suggesting that the duplication event (∼40 My ago) was transposon based. Local duplication, possibly succeding a transposon based duplication event may also have occurred, for example for MADF genes set *CG1602*, *CG1603* and *az2* and possibly for *stwl*, CG3919 pair are possible local duplicates. Interestingly, CG3919, as tested by RNAi (Table 1), does not appear to retain germ line Cell (GLC) lineage function, nor does it rescue the *brwl* mutant phenotype. In agreement, ModENCODE data also suggest low expression of CG3919 transcripts in the ovary.

What are the possible mechanisms for redundancy after gene duplication? A possible scenario is a model where MADF-BESS domain proteins are competent fo function as homo as well as heterodimers and and the Stwl-Brwl heterodimer can substitute the Brwl homodimer (Figure 8C). In the female germline, both Stwl and Brwl are expressed, thus retaining ancestral expression and functionality. Although the BESS domains have been suggested to be protein:protein intercation surfaces, there is no experimental evidence for direct molecular interaction between Stwl and Brwl in current interaction databases (Hu *et al.* 2018).

An open question in the field is the molecular function of MADF and MADF-BESS domain containing proteins. Members appear to modulate diverse processes (Ratnaparkhi *et al.* 2008; Song *et al.* 2010; Cutler *et al.* 1998; Duong *et al.* 2008; Shukla *et al.* 2014; Zimmermann *et al.* 2006; Timmerman *et al.* 2013; Casola *et al.* 2007; Barbash *et al.* 2000; Brideau *et al.* 2006) with no apparent common theme. The MADF-BESS family members may either act as transcription activators/repressors or as histone modifiers, they may also associate with different subsets of co-factors for diverse functions. Brwl, based on its functional similarity to Stwl may be a DNA binding protein and a putative chromatin regulator (Yi *et al.* 2009).

In summary, our study uncovers *brwl* as a novel player in the maintanence of GSCs in the female germline. While our data do not rule out a somatic function conlusively, our phenotypic analysis and expression data strongly suggest a possible germ cell autonomous function of the gene. *brwl* phenotypes are pleiotropic suggesting that either the protein has an early function with compounding downstream effects or it has multiple roles in the development of the cyst. This pleiotropy is reminiscent of *stwl* mutants.

Furthermore, our data point to partially redundant functions shared between *brwl* and *stwl* in promoting oocyte development in the female germline. We hypothesize that *brwl* and *stwl* likely arose as a consequence of a gene duplication event 40 My ago. We further speculate that ancestral functions are retained by both the gene products and are utilized for robust patterning of the germline cells in the developing ovaries.
